# Enfacing others but only if they are nice to you

**DOI:** 10.3389/fnbeh.2014.00102

**Published:** 2014-03-28

**Authors:** Ilaria Bufalari, Bigna Lenggenhager, Giuseppina Porciello, Brittany Serra Holmes, Salvatore M. Aglioti

**Affiliations:** ^1^IRCCS Fondazione Santa LuciaRome, Italy; ^2^Social and Cognitive Neuroscience Laboratory, Department of Psychology, Sapienza University of RomeRome, Italy; ^3^Department of Neurology, University Hospital ZurichZurich, Switzerland

**Keywords:** enfacement illusion, multisensory integration, stereotype, racial bias, interpersonal perception, social cognition

## Abstract

Experiencing tactile facial stimulation while seeing synchronous stimuli on the face of another individual induces “enfacement,” i.e., the subjective illusory experience of ownership of the other's face (explicit measure) and the attribution of the others' facial features to one's own face (implicit measure). Here we expanded previous knowledge by investigating if the tendency to include the other into one's own representation is influenced by positive or negative interpersonal attitudes derived either from consolidated socio-cultural stereotypes or from newly acquired, short-term individual interactions with a specific person. To this aim, we tested in Caucasian white participants the enfacement with a white and a black confederate, before and after an experimental procedure inducing a positive or negative perception of each of them. The results show that the subjective experience of enfacement with in- and out-group others before and after the manipulation is similar. The bias in attributing other's facial features to one's own face after synchronous stroking was, instead, dependent on whether the other person was positively perceived, independently of his/her ethnicity. Thus, we show that realistic positive face-to-face interactions are more effective than consolidated racial biases in influencing the strength of self-attribution of another persons' facial features in the context of multisensory illusions. Results suggest that positive interpersonal interactions might powerfully change the plasticity of self-other representations.

## Introduction

The sense of bodily self is based on the integration of congruent spatio-temporal multisensory information (Tsakiris, 2010) and is thought to be fundamental for higher forms of self-identity and consciousness (Gallagher, 2000).

Recent research has shown that the bodily self is much more malleable than usually assumed since relatively simple interpersonal synchronous multisensory stimulation can blur perceptual self-other boundaries. After experiencing tactile stimulation on the body while observing similar synchronous stimuli on the same body part of another individual, participants self attribute the observed body parts (e.g., rubber hand illusion) (Botvinick and Cohen, 1998) or even the full body (Lenggenhager et al., 2007; Petkova and Ehrsson, 2008). Such illusory embodiment is accompanied by physiological changes in the actual body, such as lowered body temperature (Moseley et al., 2008; Salomon et al., 2013), altered immunological responses (Barnsley et al., 2011), variations in tactile and pain thresholds (Hänsel et al., 2011). Moreover, the brain circuit coding anxiety and interoceptive awareness reacts to threats to the synchronously stimulated body as when the person's real hand is threatened (Ehrsson et al., 2007). Furthermore, increasing evidence shows that the illusory embodiment of a full body or a single body part might even alter attitudes toward others, such as implicit racial biases (Farmer et al., 2012; Peck et al., 2013).

Here, we focus on the face, since it is the most important and distinctive feature of our own body and of personal identity. The face also relays crucial information about others' mental and emotional states, which are fundamental for both social interactions and interpersonal perception (Todorov et al., 2013).

Recent research has shown that synchronous stroking of one's own and another person's face causes illusory embodiment of the face of the other person, an effect we named “enfacement” (Sforza et al., 2010). In the synchronous stimulation condition, the subjective feeling of ownership and referral of touch (explicit measure of the illusion) is accompanied by self-attribution of the other person's facial features in self-recognition and self-other discrimination tasks (implicit measure of the illusion) (see Tsakiris, 2008; Sforza et al., 2010; Tajadura-Jiménez et al., 2012a,b). This evidence led researchers to hypothesize that synchronous interpersonal stimulation induces plastic changes in the self-face representation (Tajadura-Jiménez et al., 2012a,b). Indeed, the visual representation of one's own face is built upon accumulating congruent multisensory experiences e.g., by matching one's sensorimotor experience with the sensorimotor behavior of the object (the face) seen in a mirror (Rochat, 2003; Tsakiris, 2010; Tajadura-Jiménez et al., 2012b). Thus, the detection of matching multisensory stimuli between one's own and another's face (placed in front of us) allows us to incorporate the facial features of the other into our own self-face representation.

Moreover, similarly to the rubber hand illusion, it has been shown that experiencing synchronous interpersonal stimulation with another person may also change interpersonal perception by increasing closeness, attraction, and perceived similarity toward that specific person (Paladino et al., 2010; Tajadura-Jiménez et al., 2012a).

Thus, previous literature suggests that self-representation is inherently plastic, since experiencing multisensory congruence with another person may blur perceptual self-other borders and induce bodily (i.e., inclusion of other's body) and conceptual (i.e., adoption of other's attitudes and psychological traits) self-other merging.

Here, we expanded previous knowledge by investigating if the tendency to include the other into one's own representation is influenced by positive or negative interpersonal attitudes derived from consolidated socio-cultural stereotypes or from newly acquired, short-term individual interactions.

For example, when approaching a new person, our behavior is influenced by first sight impressions, social categorizations and stereotypes that seem to be automatic and unavoidable (Cosmides et al., 2003; Degner and Wentura, 2010). Among these, group membership has shown to be a dominant factor (Brewer, 1979). People show an in-group favoritism and out-group derogation bias, i.e., tend to perceive the in-group members as more similar and evaluate them more positive than out-group members (for a review see Hewstone et al., 2002). Ethnicity is one of the strongest factors for stereotyping (Stangor et al., 1992) and plays an important role in various processes of overlapping self-other representations such as sensorimotor and affective resonance for others' actions and sensations (Avenanti et al., 2010; Liew et al., 2011; Azevedo et al., 2012), mimicry (Bourgeois and Hess, 2008), and visual enhancement of touch (Serino et al., 2009; Fini et al., 2013).

Thus, we tested whether racial group membership may modulate enfacement and if this effect depends upon implicit and explicit racial biases. To this aim, we measured implicit and explicit racial attitudes of white Caucasian female participants undergoing the enfacement paradigm [i.e., visuo-tactile synchronous (illusory condition) and asynchronous (control condition) stroking] with in-group (i.e., white) and out-group (i.e., black) partners (who were confederates of the experimenter). As sensorimotor sharing is usually stronger among ingroup members, we expected the enfacement to be stronger for ingroup (i.e., white) vs. outgroup (i.e., black) individuals.

However, despite the well-known influence of racial stereotypes on various emotional and cognitive processes, short-term interactions establishing a sense of connectedness between individuals may mediate interpersonal links and even overrule group-based stereotypes (Kurzban et al., 2001). Connectedness also increases mimicry (van Baaren et al., 2009) while felt likability (Sobhani et al., 2012) intimacy (Mazzola et al., 2010), similarity (Désy and Théoret, 2007) and perceived fairness (Singer et al., 2006) of the observed person modulate activity in brain regions that code for self and other's actions, sensations, emotions (Bufalari and Ionta, 2013) and motor behavior in dyadic interactions (Sacheli et al., 2012).

Thus, we additionally addressed the question if experimentally induced positive or negative interpersonal perception affects the enfacement effect. To this aim, we adapted a paradigm that has been previously shown to effectively change the interpersonal relationship toward a specific person (i.e., the self-esteem threatening paradigm, Caprara et al., 1987; Sacheli et al., 2012). We provided participants with manipulated false positive or negative feedback about the first impression the white and black confederate gave of them. Thus, the enfacement strength was compared before and after participants' received the feedback. We hypothesized that participants would exhibit stronger enfacement with the confederate who liked them (hereafter named “positive partner”), and weaker enfacement with confederate who disliked them (hereafter named “negative partner”), as indicated by positive/negative first impression judgments.

Recent research demonstrates that manipulating social context, cognitive strategies and intergroup relationships can diminish the extent to which race is encoded (Kurzban et al., 2001) and modulate brain activity related to racial biases (Wheeler and Fiske, 2005; Van Bavel et al., 2008; Sheng and Han, 2012). We explored the link between these variables by testing whether induced positive or negative perception of in-group vs. out-group individuals may override any possible enfacement differences driven by group membership. We hypothesized that experimentally induced interpersonal perceptions could increase facial embodiment of positive out-group members and conversely decrease facial embodiment of negative out-group members.

In order to ensure that the interpersonal manipulation was effective in changing the perception of the confederates, we measured the perceived attractiveness of the confederates before and after the participants received the first impression feedback. We expected that receiving positive vs. negative feedback would respectively increase and decrease the perceived attractiveness of the partner. This would be in keeping with social psychology and neuroscience studies showing people tend to like those who like them (Lowe and Goldstein, 1970; Aronson et al., 2010) and to perceive people with favorable personality traits as more attractive (Lewandowski et al., 2007).

Also, we tested whether inducing positive or negative interpersonal perception of specific in- and out-group members could modify participants' implicit racial attitudes toward the group the confederate belonged to. To this aim, we administered the IAT not only before but also after the interpersonal perception induction, at the end of the whole experiment.

Thus, the present experimental set-up allowed us to explore, in an ecologically valid but still well-controlled way, whether enfacement depended on positive vs. negative interpersonal perception, either defined by established socio-cultural stereotypes or by the momentary liking of the other persons.

## Methods

### Participants

Twenty-seven, normal or corrected-to-normal sighted Caucasian females (*M* = 23.9 years, *SD* = 2.9) interacted with both a black and a white female confederate. Because attractiveness plays a role in the enfacement illusion (Sforza et al., 2010), the two confederates were selected on the basis of a preliminary study in which an independent sample of female participants judged them as equally attractive {paired *t*-test [*T*_(9)_ = 1.854, *p* = 0.1]}. All the participants provided their informed consent. All were naïve to the purpose of the study, debriefed and reimbursed for their participation. The study was approved by the local Ethics Committee and was conducted according to the Helsinki declaration.

### General procedure

**Figure 1:**
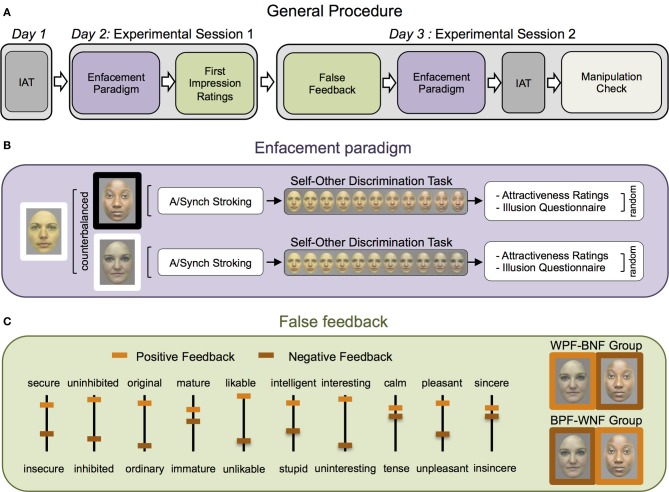
**Experimental procedure. (A)** Illustration of the general procedure, which was completed in three different sessions separated by about a week's time. Participants performed a preliminary session (where their face pictures, IAT, and personality measures were taken) and two experimental sessions during which the enfacement was induced with a black and a white confederate before and after the interpersonal perception was manipulated. **(B)** The enfacement paradigm included synchronous (illusory condition) and asynchronous (control condition) stroking of one's own and a black or white partner's face, each followed by: (i) the self-other discrimination task (rating on a 0–100 VAS how much each of the 50 images extracted from the morphing continuum between the self and other face was like their own face, with 0 = “not me at all”; 100 = “completely me”); and in random order: (ii) the attractiveness rating; and (iii) the Illusion questionnaire. **(C)** To vary the interpersonal perception in the second session, false-feedback about the first impression of both the black and the white partner was given to the participants. Depicted are the globally positive (orange) and negative feedback (brown) on the ten bipolar adjectives-VAS ratings. One group of participants received positive feedback from the white (WPF) and negative feedback from the black (BNF) partner, the other group the opposite.

An overview of the general experimental procedure is provided in Figure 1A. The study was composed of three different sessions separated by about a week's time. Participants performed a preliminary session during which pictures of their face, the race version of the Implicit Association Test (IAT), and personality measures were taken. Following, there were two experimental sessions in which the enfacement was induced with both the black and white confederate before and after the interpersonal perception was manipulated using the self-threatening paradigm by Caprara et al. (1987). The experiment was run with E-Prime software (v1.1, Psychology Software Tools, Inc., Pittsburgh, PA) on an IBM compatible computer.

#### Visual stimuli

Participants' pictures were edited using Adobe Photoshop® 7.0 software to remove external features (hair, ears) and create a uniform gray background. Then, pictures were separately morphed with the black and white confederates' faces using Abrasoft FantaMorph® 4.0 software at steps of 2%, thus obtaining 50 individual images from the morphing continuum (as in Sforza et al., 2010), whose end points were the participant's face and the other white/black person's face.

#### Enfacement paradigm

Each experimental session consisted of two runs containing the enfacement paradigm with a white or a black confederate, in counterbalanced order (Figure 1B). Participants sat facing the confederates about 150 cm apart wearing a rigid paper funnel around the eyes, which blocked the lateral view of the experimenter who touched participants' cheeks with two identical paintbrushes. Both confederates and participants were asked to concentrate on the other person's face while the two faces were manually touched synchronously (illusory-condition) or asynchronously (control-condition). Strokes and taps were made irregular and unpredictable in order to enhance the illusion (Mohan et al., 2012). In the synchronous condition, they were given in exact spatial and temporal synchrony while in the asynchronous condition a delay between the strokes on the two faces was introduced. Stimulation type order was counterbalanced across participants.

***Explicit measure of the enfacement: self-report questionnaire on the phenomenological experience.*** The subjective phenomenological experience of the illusion was investigated by asking participants to fill out a questionnaire, adapted by Sforza et al. (2010) from the first seminal study on the rubber hand illusion (Botvinick and Cohen, 1998). The questionnaire, filled out at the end of each a/synchronous stroking block, consisted of the following eight statements investigating specific perceptual experiences:

STATEMENT 1: It seemed as if I were feeling the touch of the paintbrush in the location where I saw the other's face touched.
STATEMENT 2: It seemed as though the touch I felt was caused by the paintbrush touching the other's face.
STATEMENT 3: I felt as if the other's face was my face.
STATEMENT 4*:* It felt as if my face were drifting toward the other's face.
STATEMENT 5*:* It seemed as if I might have more than one face.
STATEMENT 6*:* It seemed as if the touch I was feeling came from somewhere between my own face and the other's face.
STATEMENT 7*:* It appeared as if the other's face were drifting toward my own face.
STATEMENT 8: The other's face began to resemble my own face, in terms of shape, skin tone, or some other visual feature.

Items 1–3 and 8 describe the experience of the illusion in its components of referred sensation, self-identification and similarity with the observed face (Botvinick and Cohen, 1998; Tajadura-Jiménez et al., 2012b). The other questions were the control items (Botvinick and Cohen, 1998). Participants indicated their response on a VAS ranging from completely false to completely true.

***Implicit measure of the enfacement: self-other discrimination task.*** A sensitive measure of the self-other discrimination ability was used to tackle changes in the visual representation of the self-face induced by the synchronous interpersonal multisensory stimulation Self-attribution scores of self-other morphed faces were used as an implicit measurement of the enfacement effect (as in Sforza et al., 2010). Immediately after the visuo-tactile stimulation, participants performed the self-other discrimination task by rating on a 0–100 visual analog scale (VAS) how much each morphed face on the screen was like their own face (0 = “not me at all”; 100 = “completely me”). Each stroking type was presented in two subsequent mini-blocks, each one containing 2 minutes of stroking followed by self-other discrimination of 25 images selected from the morphing continuum. A different set of 25 images was presented in each of the two mini-blocks. Images in each set were pseudo-randomly selected such that 2 or 3 images were selected from each morphing category (categories were defined as intervals of 10% of morphing continuum).

#### Interpersonal manipulation

A cover story was used, telling participants that they were also recruited for a study on first impressions. Following the self-threatening paradigm (Caprara et al., 1987), at the end of the first experimental session, we asked confederates and real participants to judge how much ten bipolar adjectives described their partners. At the beginning of the second experimental session, before the confederates' arrival, participants were shown what both confederates said about them and were asked to express how much (on a 0–100 VAS, with 0 = “completely disagree” and 100 = “completely agree”) they agreed with the received evaluations. The feedback was manipulated: one group received globally positive feedback from the white confederate and negative feedback from the black confederate (White Positive Feedback group), while the other received the opposite pattern (Black Positive Feedback group) (see Figure 1C, where adjectives used in the experiment have been translated to English).

#### Measures of the effectiveness of the interpersonal manipulation: attractiveness ratings

To check if perception of confederates changed after the interpersonal manipulation, attractiveness ratings of the other were collected (on a 0–100 VAS, where 0 corresponded to “very low” and 100 to “very high”) before and after the interpersonal manipulation. Studies indicate that enfacement increases the enfaced other's perceived attractiveness, and is in turn influenced by it (Paladino et al., 2010; Sforza et al., 2010; Tajadura-Jiménez et al., 2012a). Therefore, we collected attractiveness ratings for each testing session after each synchronous and asynchronous stroking block.

#### Measures of the effectiveness of the interpersonal manipulation: implicit and explicit racial biases

To check if participants' implicit racial bias changed after the interpersonal manipulation, the race version of the implicit association test (IAT, Greenwald et al., 1998) was administered to participants before and after the interpersonal manipulation, i.e., in the preliminary session (IAT-pre), and at end of the second session (IAT-post). During the racial IAT participants were asked to press as fast and accurate as possible the same button for certain faces (white or black) and words (positive or negative). For example, participants had to press the same key for white faces and positive adjectives in one condition and the same key for black faces and positive adjective in the other (please see Supplementary Material for a complete description of the IAT procedure). The implicit bias is calculated from the differences in speed and accuracy between the pairing of white faces with positive words, and black faces with negative words vs. the pairing of white face with negative words, and black faces with positive words. We calculated the two IAT scores using the algorithm recommended by Greenwald et al. (2003), taking into account the average reduction of scores in the second measurement. Greater racial bias in favor of white is indicated by higher (greater than 0) IAT scores [i.e., longer reaction times and lower accuracies in associating black faces, as compared to white faces, with positive (compared to negative) words].

Even if racial bias is more readily observed at an implicit level, (Greenwald et al., 1998; Ito and Bartholow, 2009), we also checked for the presence of explicit racial biases with an *ad-hoc* interview, adapted from Avenanti et al. (2010). The nine questions and associated subjective ratings (on 0–100 VAS, with 0 = “completely disagree” and 100 = “completely agree”) are listed in Table 1.

**Table 1:** **The 9-items of the *ad-hoc* interview on explicit racial biases are shown below**.

**Questions**	**Mean ± *SD***
Do you think that, even if Italians and Africans become friends, they will never feel completely at ease in their interactions?	7.96 (± 8.9)
Would you be bothered by the event that a member of your family has a child with physical features (e.g., color of the skin) different from yours?	8.92 (± 20.05)
Do you think that Africans take jobs that Italians deserve?	10.08 (± 14.3)
Do you think that Africans and Italians are comparable in terms of their honesty?	71.77 (± 30)
Would you be keen to have an intimate relationship with an African?	72.88 (± 22.9)
Would you be against a member of your family married to an African of comparable economic status?	3.58 (± 5.1)
Would it be a problem for you to have an African boss?	7.81 (± 15.2)
Is your family of Italian origin?	94.88 (± 19.3)
Do you frequently meet African people (relatives, friends, or colleagues)?	49.12 (± 32.3)

Means and standard deviations are reported.

#### Measures of participant empathic traits

Enfacement strength shows a positive correlation with empathic traits (Sforza et al., 2010). Thus, to control for the possible contribution of empathy to the hypothesized effects of ethnicity/interpersonal manipulation on the enfacement, we measured participants' empathic traits. In the preliminary session, participants completed the Interpersonal Reactivity Index (IRI), (Davis, 1980), a personality questionnaire comprising 28 items to be rated on a five-point Likert scale. The IRI consists of four subscales that measure different aspects of trait-reactivity to others. Specifically, Fantasy Scale (FS) and Perspective Taking (PT) measure cognitive empathy, while and Empathic Concern (EC), and Personal Distress (PD) measure emotional empathy.

#### Manipulation check

Finally, to check if participants believed the cover story, before debriefing they were asked to respond whether they believed the first-impression was a real study and if they were either offended or pleased by the negative and positive feedback (on 0–100 VAS, with 0 = minimally, and 100 = maximally).

### Data analyses

#### Preliminary analyses

To exclude the possible influence of spurious factors on the changes induced in the enfacement illusion by our independent variables (ethnic in/out-group membership; induced positive/negative interpersonal perception), we ran a series of preliminary analyses to ensure that the two groups did not differ in variables that could play a role in the enfacement.

***Racial biases.*** Statistical analyses confirmed that the two groups (positive feedback from the black, positive feedback from the white) did not initially differ in implicit (see Main Results section) racial bias. Also, their explicit racial biases (see Table 1 for items) did not differ as shown by a 2 × 9 mixed-model ANOVA (Social Manipulation; Item) which revealed only a significant main effect of the Item [*F*_(8, 168)_ = 85.59, *p* < 0.000] but no significant main effect of the Social Manipulation nor an interaction (all *F*-values < 1.73, all *p*s > 0.094).

***Empathy traits.*** Due to the known interaction between empathic traits and enfacement strength (Sforza et al., 2010), we checked that the two groups did not show differences on the IRI questionnaire. A mixed-model 2 × 4 ANOVA (Interpersonal Manipulation; IRI subscales) confirmed the lack of differences, since there was a main effect of the IRI subscales [*F*_(3, 66)_ = 12.63, *p* = 0.000; EC (21.08 ± 3.55), PD (13 ± 5.05), PT (18 ± 4.64), FS (17.08 ± 4.77)] and no significant main effect of the interpersonal manipulation nor an interaction (all *F*-values < 1.31, all *p*s > 0.265).

***Reactions to the interpersonal manipulation.*** To ensure that both groups equally believed the manipulation, a *t*-test comparison of the VAS scores indicating how much participants believed the cover story was calculated. No significant difference was found [*T*_(22)_ = 1.42, *p* = 0.17]. Moreover, the two groups felt equally offended and pleased from receiving the negative and the positive feedback from both the white or the black confederate. A mixed model 2 × 2 ANOVA [Interpersonal manipulation (White Positive Feedback group; Black Positive Feedback group); Items (“How much did you feel offended by the feedback?”; “How much did you feel pleased by the feedback?”)] revealed no significant main or interaction effect (all *F*-values < 2.52, all *p*s > 0.127).

***First impression ratings on confederates' personality and attractiveness.*** The two groups perceived the two confederates similarly both in terms of physical beauty (attractiveness) and of personality traits (first impressions). Results of the statistical analysis on perceived attractiveness as measured in the first session are embedded in the Main Results section.

Results of the mixed model ANOVA 2 × 2 × 10 with Race and Adjective as within subjects and Social Manipulation as between subjects factors performed on the first impression ratings showed that participants in the two groups similarly judged the black and white confederates. There was only a main effect of the Adjectives [*F*_(2, 198)_ = 4.61, *p* < 0.000] but no other significant main (all *F*-values < 1.32, all *p*-values > 0.263) or interaction (all *F-values < 1.73*, all *p*-values > 0.084) effects.

#### Main analyses

***Manipulation check.*** First, we tested the efficacy of the interpersonal manipulation, e.g., how much participants believed they were involved in a real interaction and were pleased and offended from receiving positive and negative feedback from the white and/or the black confederate. Participants' scores of the three manipulation check items were tested using one sample *t*-test against a score of 50 (which corresponds to a “neither agree nor disagree” judgment on the 0–100 VAS scale).

***Explicit measures of the enfacement (questionnaire items).*** Subjective ratings were analyzed using mixed-design ANOVAs with Interpersonal Manipulation (White Positive Feedback; Black Positive Feedback) as between subjects factor and Race (White; Black), Session (1st; 2nd), Stroking (Synchronous; Asynchronous) and Item [only for the questionnaire ratings: illusion-relevant (Q1–Q3, Q8), illusion-irrelevant (Q4–Q7)]; see e.g., Lenggenhager et al., 2012 for a similar approach) as within-subjects factors. When a significant fourth-way interaction was found, *post-hoc* ANOVAs were run separately for each session.

***Implicit measure of the enfacement (self-other discrimination task).*** The whole set of 2% rating values of the morphed images was fitted into a four-parameter sigmoid statistical model [which was based on the Boltzmann equation: y0 = A1 − A2/[1 + e^(x − x0/dx)^] + A2] for each subject and experimental condition. Appropriateness of the model was demonstrated for all the conditions at individual level (all Radj ≥ 0.361; *p*s < 0.01) and the X0 values were extracted for each subject and condition. X0 value corresponds to the physical percentage of self-other morph values on the abscissa when subjective ratings were 50% on the ordinate. Three participants had X0 outlier values (±2.5 *SD*) in one or more experimental conditions and were excluded from all statistical analyses. Then, self-attribution indices (X0 values) were analyzed using mixed-design ANOVAs with Interpersonal Manipulation (White Positive Feedback; Black Positive Feedback) as between subjects factor and Race (White; Black) Session (1st; 2nd), Stroking (Synchronous; Asynchronous) as within-subjects factors. When a significant fourth-way interaction was found, *post-hoc* ANOVAs were run separately for each session.

***Attractiveness ratings.*** Attractiveness modulations as a function of interpersonal manipulation, ethnic membership or enfacement, were tested with a 2 × 2 × 2 × 2 ANOVA with between subject factors Interpersonal Manipulation and within subjects factors Race, Session, Stroking.

***Implicit racial bias.*** Modulations of implicit racial biases as due to the interpersonal manipulation were tested with a 2 × 2 [Interpersonal Manipulation and Time (pre-; post-manipulation)] ANOVA performed on the IAT scores.

For all the above mentioned analyses, when significant main or interaction effects were found, Duncan *post-hoc* comparisons were used. All data were analyzed with STATISTICA 7 software (StatSoft, Tulsa, OK, USA) and the significance level was set at *p* = 0.05.

## Results

### Main results

#### The interpersonal manipulation was effective and participants believed the cover story

Agreement scores for the three manipulation check items were significantly higher than 50, Indeed, participants believed the first-impression study was real [“believability” scale: (93.82 ± 14.66) (mean ± *SD*), *t*_(1, 23)_ = 14.646, *p* = 0.000]. Participants also felt offended [(62.30 ± 26.53), *t*_(1, 23)_ = 2.271, *p* = 0.033] and pleased [(67.53 ± 32.87), *t*_(1, 23)_ = 2.612, *p* = 0.016] by the partner's negative and positive judgments, since their scores were significantly higher than 50, and with equal intensity [offended vs. pleased: *t*_(1, 23)_ = −0.695, *p* = 0.494].

#### Explicit and implicit measure of the enfacement effect

**Figure 2:**
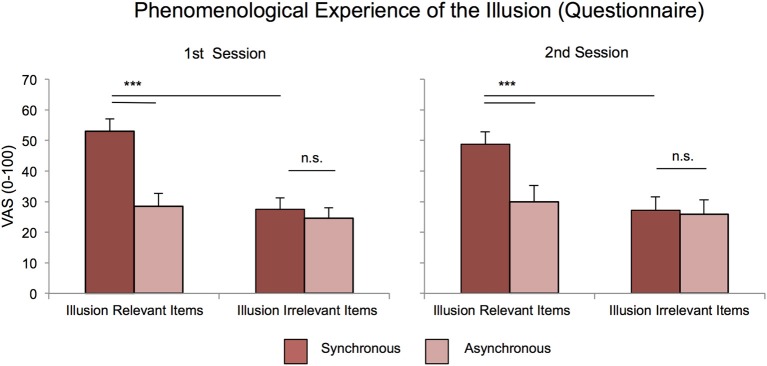
**Explicit measures of enfacement**. At the explicit level, participants reported higher scores after synchronous with respect to asynchronous stroking only in the items describing the experience of the illusion, in both sessions and independently of group membership and interpersonal manipulation. Agreement scores on a 0–100 VAS scales are shown (mean ± *SE*). Results of the significant 2-way interactions between Stroking and Questionnaire Items are reported for the first (left panel) and second (right panel) session. [Asterisks indicate significant differences (^***^Stands for *p* ≤ 0.001)].

***The explicit measure of the illusion: the enfacement strength does not depend on the other's ethnic membership and positive/negative perception.*** Results show the illusion was present in both sessions and independently of ethnic membership and interpersonal manipulation (Figure 2).

Results of the 2 × 2 × 2 × 2 × 2 ANOVA revealed a main effect of Stroking [*F*_(1, 22)_ = 17.53, *p* < 0.000, η^2^_*p*_ = 0.444], Item [*F*_(1, 22)_ = 20.26, *p* < 0.000, η^2^_*p*_ = 0.479], a significant Stroking × Item interaction [*F*_(1, 22)_ = 20.78, *p* < 0.000, η^2^_*p*_ = 0.486], a significant Race × Session × Interpersonal Manipulation interaction [*F*_(1, 22)_ = 8.52, *p* = 0.01, η^2^_*p*_ = 0.279] and a significant 4-way interaction of Interpersonal Manipulation, Session, Race, and Stroking [*F*_(1, 22)_ = 6.51, *p* = 0.02, η^2^_*p*_ = 0.228].

Then, results of the 2 × 2 × 2 × 2 (Interpersonal Manipulation; Race; Stroking; Item) *post-hoc* ANOVAs show that there was a main effect of Item in both the first [*F*_(1, 22)_ = 20.53, *p* < 0.000, η^2^_*p*_ = 0.483] and the second session [*F*_(1, 22)_ = 14.75, *p* = 0.001, η^2^_*p*_ = 0.401], a main effect of Stroking in both the first [*F*_(1, 22)_ = 16.54, *p* = 0.001, η^2^_*p*_ = 0.429] and second session [*F*_(1, 22)_ = 11.13, *p* = 0.003, η^2^_*p*_ = 0.336] and crucially also a significant interaction between Item and Stroking in both the first [*F*_(1, 22)_ = 20.04, *p* = 0.000, η^2^_*p*_ = 0.477] and the second session [*F*_(1, 22)_ = 12.73, *p* = 0.002, η^2^_*p*_ = 0.366], with higher values after synchronous compared with asynchronous stimulations only in the illusion-related items [1st session: (53.11 ± 4.70) vs. (28.41 ± 4.81), *p* < 0.000; 2nd session: (48.79 ± 3.43) vs. (29.89 ± 5.03), *p* < 0.000; see Figure 2].

Furthermore, the ANOVA on the second session ratings revealed a significant Interpersonal Manipulation × Race interaction [*F*_(1, 22)_ = 12.16, *p* = 0.002, η^2^_*p*_ = 0.356], with higher scores for the white compared to the black found in the White Positive Feedback group [white (35.01 ± 7.63), black (26.85 ± 6.67), *p* = 0.002] but not in the Black Positive Feedback group [white (33.89 ± 6.58), black (36.75 ± 5.96), *p* > 0.24]. This effect was absent in the first session [*F*_(1, 22)_ = 0.28, *p* = 0.603].

***Implicit measure of the illusion: the enfacement strength depends on receiving positive feedback from the other independently of her group membership.*** Results of the 2 × 2 × 2 × 2 ANOVA (Interpersonal Manipulation; Session; Race; Stroking) revealed a significant main effect of Session [*F*_(1, 22)_ = 7.48, *p* = 0.012, η^2^_*p*_ = 0.254] and a 4-way interaction [*F*_(1, 22)_ = 5.64, *p* = 0.027, η^2^_*p*_ = 0.204].

**Figure 3:**
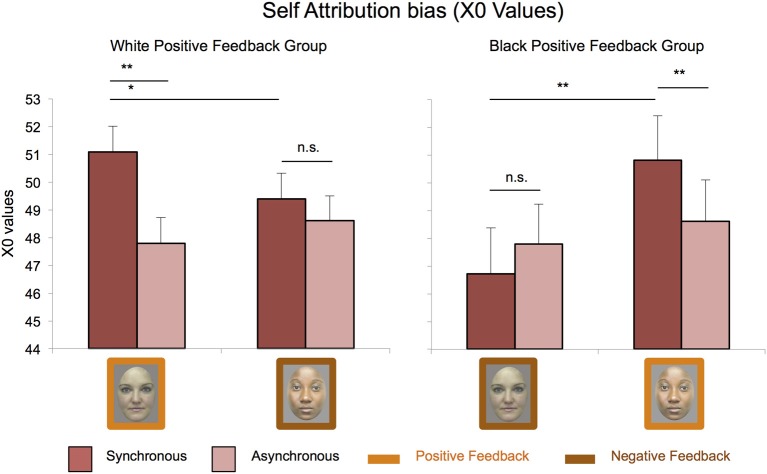
**Implicit measures of enfacement**. At the implicit level, the illusion was present as indexed by higher self-attribution scores after the synchronous compared to the asynchronous condition only for liked partners independently of their group membership. Results of the significant 3-way interaction between interpersonal manipulation, race, and stroking on X0 values (± *SE*) are shown. Asterisks indicate significant differences (^**^Stands for *p* ≤ 0.01; ^*^Stands for *p* ≤ 0.05).

The 2 × 2 × 2 (Race; Stroking; Interpersonal Manipulation) *post-hoc* ANOVA of the first session did not reveal any significant main or interaction effect (all *F*-values < 2.963, all *p*s > 0.099). The 2 × 2 × 2 (Race; Stroking; Interpersonal Manipulation) *post-hoc* ANOVA of the second session revealed a significant 3-way interaction [*F*_(1, 22)_ = 15.16, *p* < 0.001, η^2^_*p*_ = 0.408] (Figure 3). Duncan *post-hoc* analyses showed stronger self-attribution scores after synchronous vs. asynchronous stroking for the white face in the White Positive Feedback group [(51.08 ± 1.37) vs. (49.1 ± 1.31), *p* = 0.01], and for the black face in the Black Positive Feedback group [(50.82 ± 1.60) vs. (48.6 ± 1.50), *p* = 0.01]. Furthermore, synchronous stroking of the white positive face led to higher self-attribution than synchronous stroking of the black negative face [(49.39 ± 1.00), *p* = 0.02] in the White Positive Feedback group. Similarly, synchronous stroking of the black positive face led to higher self-attribution than synchronous stroking of the white negative face [(46.72 ± 1.68), *p* = 0.00] in the Black Positive Feedback group. No significant differences emerged between synchronous and asynchronous stroking for the white or the black face that gave negative feedback (all *p*s > 0.12).

**Figure 4:**
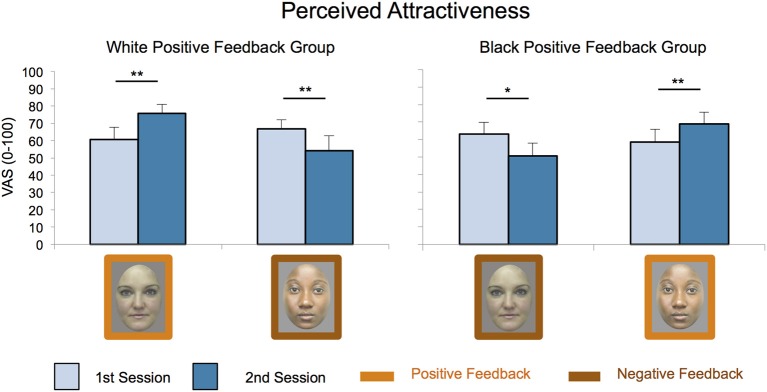
**Attractiveness ratings attributed to the partners**. Attractiveness ratings (on a 0–100 VAS scale with 0 = “minimally attractive” and 100 = “maximally attractive”) increased after participants received positive feedback and decreased after they received negative feedback from the partner, independently of their group membership. Results of the significant 3-way interaction between Interpersonal Manipulation, Race and Session are shown. Depicted are mean and standard errors. Asterisks indicate significant differences (^**^Stands for *p* ≤ 0.01; ^*^Stands for *p* ≤ 0.05).

***Perceived attractiveness increases after receiving a positive feedback from the other independent of her race.*** 2 × 2 × 2 × 2 ANOVA (Interpersonal Manipulation; Race; Session; Stroking) confirmed that interpersonal manipulation and enfacement changed the perception of the confederates as predicted (Figure 4).

We found overall higher attractiveness ratings after enfacement induction, as shown by the significant main effect of Stroking [*F*_(1, 22)_ = 6.42, *p* = 0.019, η^2^_*p*_ = 0.226; synchronous (63.13 ± 0.32) (mean ± *SE*), asynchronous (61.44 ± 0.27)]. We also found a three-way interaction of Interpersonal Manipulation, Session and Race [*F*_(1, 22)_ = 32.76, *p* < 0.000, η^2^_*p*_ = 0.598]. *Post-hoc* tests showed that attractiveness increased for the confederate who gave positive feedback to the participants and decreased for the confederate who gave a negative feedback, independent of her ethnicity.

Indeed, the attractiveness of the white confederate increased while attractiveness of the black decreased in the White Positive Feedback group in the second compared to the first session [White: (60.58 ± 7.32) vs. (75.62 ± 5.24), *p* = 0.005; Black: (66.92 ± 5.29) vs. (54.27 ± 7.92), *p* = 0.016]. Similarly, in the Black Positive Feedback group attractiveness of the black confederate increased, while attractiveness of the white decreased [Black: (56.41 ± 7.49) vs. (68.77 ± 6.78), *p* = 0.045; White: (63.09 ± 6.68) vs. (50.63 ± 7.32), *p* = 0.017]. Furthermore, in the second session the white face was judged as more attractive than the black face (*p* = 0.000) in the White Positive Feedback group, and the black face was considered as more attractive than the white face (*p* = 0.001) in the Black Positive Feedback group. No such differences were found in the first session [White Positive Feedback: white (60.58 ± 7.32), black (66.92 ± 5.29); Black Positive Feedback: white (63.09 ± 6.68), black (58.41 ± 77.49); all *p*s > 0.187], thus showing that participants belonging to the two groups perceived the black and white confederates as equally attractive before receiving the interpersonal manipulation.

***Implicit racial bias does not vary after receiving interpersonal manipulation.*** The induction of positive or negative perception of the black confederate did not respectively decrease or increase participants' implicit racial bias. The 2 × 2 ANOVA revealed no significant main or interaction effects (all *F*-values < 1.17, all *p*s > 0.29). It should be noted that participants' mean pre-IAT *D* score was 0.534 (*SD* = 0.326, range: 0.11–1.368) meaning that our participants showed an implicit pro-white bias at the beginning of the two following experimental sessions, with considerable between-subjects variability.

## Discussion

Both neuroscientific and philosophical theories argue that the sense of self is importantly anchored to the neural mechanisms representing the body (Damasio, 1994; Berlucchi and Aglioti, 1997; Bermudez et al., 1998; Botvinick and Cohen, 1998; Damasio et al., 2000; Gallagher, 2000, 2005; Metzinger, 2003; Zahavi, 2005; Legrand, 2007). Yet, evidence is rising that the bodily self is more malleable than previously assumed and influenced by moment-to-moment online integration of multisensory input (Carruthers, 2008). In line with this idea, empirical research has shown that relatively simple multisensory stimulations can alter the sense of self. It has been shown, for example, that another person's bodily features can be misattributed to one's own bodily self (e.g., Botvinick and Cohen, 1998; Lenggenhager et al., 2007; Tsakiris, 2008; Sforza et al., 2010), and also that perception of one's own body (in terms of form and size; Normand et al., 2011) and consequently the size of the objects in the surrounding world (van der Hoort et al., 2011; Banakou et al., 2013) may change according to the form and size of the synchronously stimulated virtual body.

Thus, the multisensory stimulation approach bears a unique possibility to study the plasticity of the bodily self and its interaction with social cognition. Importantly, self-other sensorimotor sharing may be fundamentally linked to human social and prosocial behavior (Avenanti et al., 2010).

Despite the growing interest in understanding the bodily representation of the self and others and its plasticity, very little is known about how self-other merging with a real other person shapes the social relationship between the two. Two important studies revealed that self-other merging may influence the link between the self and other (Paladino et al., 2010; Tajadura-Jiménez et al., 2012a). Our results show for the first time how self-other merging is modulated by positive or negative attitudes toward others. We specifically assessed and manipulated attitudes defined by enrooted, group-based categorization processes (i.e., ethnic in-/out-group membership) and, on the other hand, attitudes defined by short-term, real-life interactions.

Crucially, the present results, obtained through a well-controlled but still ecologically valid experimental set-up, show that the interpersonal perception derived from socio-cultural biases does not influence the enfacement. Conversely, the perception based on individual interactions does. It is important to note, that at an explicit, conscious level, white participants report to feel the illusion similarly for both black and white confederates (both before and after receiving a positive or negative feedback from them). The more subtle implicit measure of enfacement reveals, instead, that participants include in the visual representation of their face, only the individual who provided a positive view of their personality. The finding that implicit and explicit measures of self-other confusions can be dissociated is in keeping with previous studies (Rohde et al., 2011).

We believe that these findings bear important insights on the interplay of sensorimotor sharing, self-other merging and social cognition, which might be relevant for both neuroscience and the broader social sciences. In particular, our results support the notion that the bodily self-representation is not only formed and maintained through one's own personal and private experiences, but is also plastically modulated by social variables.

### Individuals belonging to a different race (ethnic group) can still be enfaced

During social interactions, humans are extremely prone to categorize and divide others in a “us vs. them” fashion (Tajfel, 1981; Amodio, 2008). Interestingly, people not only distinguish the others from the self, but spontaneously classify others into in-groups and out-groups, according to socially relevant categories, such as race, age, gender as well as by first sight impressions, which are generally automatic and unavoidable (Cosmides et al., 2003; Degner and Wentura, 2010). Ethnicity represents a powerful, salient and very fast (in the range of milliseconds) cue for group membership, social categorization and evaluation, especially in the absence of other affiliation factors (see Kurzban et al., 2001).

Behavioral and neuroimaging techniques have been employed to investigate the role of group membership in modulating shared bodily representations. These studies demonstrated that the ethnicity dimension plays an important role in several instances of self-other representations (Bourgeois and Hess, 2008; Serino et al., 2009; Avenanti et al., 2010; Liew et al., 2011; Azevedo et al., 2012). Interestingly, a very recent study found that illusory embodiment through multisensory stimulation might alter race-specific effects on visual enhancement of touch depending on the participant's racial bias (Fini et al., 2013).

In view of this, we expected stronger enfacement effect for the in-group members. Yet, contrary to our hypothesis, our data indicate that both races were equally enfaced at the explicit level (phenomenological experience of the illusion evaluated by using the questionnaires from Sforza et al., 2010) and implicit level (self attribution scores at the self-other discrimination task). This is in line with findings from studies showing that black and white hands or virtual bodies can be similarly included in the body representation of white individuals (Farmer et al., 2012; Peck et al., 2013). The fact that race does not influence enfacement may be even more surprising since the face is seemingly most relevant for one's own and another person's identity (Sforza et al., 2010) and, in principle, is very relevant for distinguishing the self from others, and in-group from out-group members.

However, we note that although robust, this finding cannot be generalized to societal groups at large. The participants involved in our study, for example, were all university students with mild implicit in-group bias. It is entirely possible that participants with extremely high racial bias and/or negative life-experiences with out-group ethnic members could show different results.

### Enfacement is influenced by the previous behavior of the interacting partner

While racial group membership did not influence the enfacement, positive/negative interpersonal perception linked to the positive/negative evaluation of the person after a face-to-face interaction, strongly influenced self-other merging at the implicit level, as measured by self-attribution ratings. It has previously been shown that experiencing the enfacement effect can affect social perception by increasing perceived similarity (Paladino et al., 2010). Yet, this is the first study showing the other side of the coin, namely that the perception of the other person can influence the enfacement effect. Persons who previously behaved nicely toward the participants (by providing positive first impression judgments of them) are more readily enfaced, leading to stronger self-other misattribution.

An influence of both positive and negative interpersonal perception has been shown in several instances of self-other affective and sensori-motor sharing (Singer et al., 2006; Désy and Théoret, 2007; Sacheli et al., 2012; Sobhani et al., 2012; for a review see van Baaren et al., 2009; Bufalari and Ionta, 2013). We expand this notion by showing that our vis-à-vis social perception of a partner modulates also the notion of one's own bodily borders. In fact, interpersonal visuo-tactile synchronous stroking causes bodily self-other merging, i.e., a misattribution of the other person's facial features to the self. Contrary to self-other sharing, self-other merging seems to be modulated only by positive interpersonal perception. An important difference between sensorimotor self-other sharing (e.g., during empathy) and sensorimotor self-other merging (as during the enfacement) may be at the basis of this discrepancy. Sensorimotor self-other sharing sets the basis for understanding the “other” and is possibly at the basis of prosocial behavior (Singer and Lamm, 2009), but does not lead to self-other misattribution. The self-other distinction remains indeed clear. In contrast, self-other merging induced by interpersonal multisensory synchronous stroking blurs the distinction between self and others, and possibly changes the way we represent the self (i.e., the self-face: Tajadura-Jiménez et al., 2012a). While further studies will be necessary to better explain why we enface only others who are likable, we tentatively link this effect to a sort of “self-defending” strategy, i.e., we only include likable features in the representation of our self. Indeed, it is known that people tend to think of themselves as having more positive qualities and fewer negative qualities than others (Zuckerman, 1979; Taylor and Brown, 1994; Shepperd et al., 2008) and show pervasive self-serving biases in perceptual or cognitive processes to maintain and protect positive self-views (Mezulis et al., 2004).

Overall, the fact that a simple multisensory integration-related, short-lasting manipulation of interpersonal perception, can alter bodily self-representations while race does not, suggests that individual real-life interactions are more powerful than automatic group-baseXd categorization processes (i.e., ethnic in-/out-group membership). This result expands on previous studies showing that situational interactions with a specific individual are more important than established stereotypes and categorization (Kurzban et al., 2001).

In this respect, the IAT results found in our study deserve further discussion. We found that inducing positive/negative perception of a given individual changed her attractiveness independently on her ethnic group, but wasn't able to change attitudes toward the social group the confederate belonged to. Note, however, that the IAT was administered at the end of the second experimental session, after participants performed a/synchronous stroking sessions with black and white confederates. Administering the IAT just one time instead of after each stroking block was done to avoid possible learning effects due to repeating the test too many times in a limited period of time. Very recent studies showed that embodiment of black rubber hands or virtual avatars may reduce the implicit racial attitudes toward the out-group for white participants (Farmer et al., 2012; Peck et al., 2013). Thus, we cannot exclude that the lack of a significant change in the IAT scores may be due to the interfering effect of a/synchronous stroking with black/white confederates.

## Conclusion

Overall, our data suggest that at an explicit, subjective level, we can enface even dissimilar, disliked, and unfamiliar others. However, at a subtler, implicit level, the self-other merging depends on positive interpersonal perception derived from individual-based interactions. Notably, the effect seems to be at play not only for established positive perceptions (like those derived from long term friendship; Sforza et al., 2010) but also for experimentally induced momentary mutual liking. The fact that even very short-lasting social interaction can influence the amount of self-other merging suggests that social relationships are highly relevant for moment-to-moment construction of a bodily self and suggests that the plasticity of facial representation is greater than previously believed.

Thus our results may pave the way for the development of experimental paradigms for research on patients with defective self-other interactions, such as those found in autism, avoidant personality disorders and social phobias.

## Author's contribution

Ilaria Bufalari, Bigna Lenggenhager, and Salvatore M. Aglioti developed the study concept. All authors contributed to the study design. Data collection and analysis were done by Ilaria Bufalari, Bigna Lenggenhager, Giuseppina Porciello, and Brittany Serra Holmes. The manuscript was drafted by Ilaria Bufalari, Bigna Lenggenhager, Giuseppina Porciello, and Brittany Serra Holmes, and critically reviewed by Salvatore M. Aglioti. All authors approved the final version of the paper for submission.

### Conflict of interest statement

The authors declare that the research was conducted in the absence of any commercial or financial relationships that could be construed as a potential conflict of interest.
