# Supplementary material for: Enfacing others but only if they are nice to you
Source: Front Behav Neurosci. 2014 Mar 28;8:102. doi: 10.3389/fnbeh.2014.00102 (PMC3975105; doi:10.3389/fnbeh.2014.00102)
Supplement: Supplementary file 1 [file DataSheet1.PDF]

SUPPLEMENTARY MATERIALS

IAT procedure

The table shows the IAT procedure as recommended by Greenwald (Greenwald et al., 1998). Participants performed version A and B of the IAT in sequential order. Each version contained 7 blocks of trials.

According to the instructions given at the beginning of each block, a stimulus (either a picture of a face or a word) appeared in the center of the screen and participants had to press as fast and accurate as possible a right/left key on the computer keyboard (i.e., 'P' or 'Q') associated to the category the stimulus belonged to (i.e., 'White/Black', 'Positive/Negative' or combined 'White Face/Positive Word' and 'Black Face/Negative Word', depending on the block). Pairing of categories with the left/right key was reminded in each trial by showing the category labels at the right/left upper sides of the screen. For example, in Block 3 (Version A) the label 'White Face/Positive Word' was present at the upper left part of the screen, while the label 'Black Face/Negative Adjective' at the upper right part of the screen in each trial, and participants were asked to press left ('Q') key to the appearance in the center of the screen of either a white face or a positive word, and to press right ('P') key to the appearance either a black face or a negative word.

The face stimuli (visible at: http://implicit.harvard.edu/) consisted of 6 white faces (3 females, 3 males) and 6 black faces (3 females, 3 males). The word stimuli consisted of 8 positive (i.e., Pleasure, Happy, Peace, Love, Laughter, Cheer, Glory, Joy) and 8 negative words (i.e., Agony, Grief, Ugly, Failure, Horrible, Nuisance, Terrible, Wicked).

IAT scoring

The IAT scoring was calculated as recommended in (Greenwald et al., 2003).

In each participant and block, the Reaction Times (RTs) in the incorrect trials were replaced by the mean of RTs in correct trials of that block plus 600 ms. Then, the mean of 'Penalty Reaction Times (RTs)' was calculated for each block in each participant by averaging correct and incorrect (modified) RTs.

For each version (A or B) a final score was calculated as the average of two indexes, calculated as follow: 1) [(Block 6 mean Penalty RTs) – (Block 3 mean Penalty RTs)/ standard deviation of pooled Block 6 and Block 3]; and 2) [(Block 7 mean Penalty RTs) – (Block 4 mean Penalty RTs)/ standard deviation of pooled Block 7 and Block 4]. Then the final IAT score was calculated as the average of the version A and version B final scores.

	Order of the Blocks	**Version A**		**Version B**	
		Stimulus	*Correct response*	*Stimulus*	*Correct response*
**Initial target-concept discrimination**	1<br>(20 trials)	White Face<br>Black Face	Q<br>P	White Face<br>Black Face	P<br>Q
**Associated attribute discrimination**	2<br>(20 trials)	Positive Word<br>Negative Word	Q<br>P	Positive Word<br>Negative Word	Q<br>P
**Combined task I**	3<br>(20 trials)	White Face/Positive Word<br>Black Face/Negative Word	Q<br>P	White Face/Negative Word<br>Black Face/Positive Word	Q<br>P
**Combined task II**	4<br>(40 trials)	White Face/Positive Word<br>Black Face/Negative Word	Q<br>P	White Face/Negative Word<br>Black Face/Positive Word	Q<br>P
**Reversed target-concept discrimination**	5<br>(20 trials)	White Face<br>Black Face	P<br>Q	White Face<br>Black Face	Q<br>P
**Reversed combined task I**	6<br>(20 trials)	White Face/Negative Word<br>Black Face/Positive Word	P<br>Q	White Face/Positive Word<br>Black Face/Negative Word	Q<br>P
**Reversed combined task II**	7<br>(40 trials)	White Face/Negative Word<br>Black Face/Positive Word	P<br>Q	White Face/Positive Words<br>Black Face/Negative Word	Q<br>P
